# Supplementary material for: Predicting visual function by interpreting a neuronal wiring diagram
Source: Nature. 2024 Oct 2;634(8032):113–23. doi: 10.1038/s41586-024-07953-5 (PMC11446822; doi:10.1038/s41586-024-07953-5)
Supplement: Supplementary file 6 — CRF and ERF predictions for individual TmY4 and TmY9 cells. Analogous to Supplementary Data 3, but for TmY target types. Shown are the top four monosynaptic pathways, the strongest pathway passing through each of the top ten intermediary types (ranking from Extended Data Fig. 7), and the trisynaptic pathway Tm1–TmY–Dm3–TmY (see the section entitled Prediction of spatial normalization). [file 41586_2024_7953_MOESM6_ESM.zip › DataS4/TmY9qΓèÑ/720575940628219665.pdf]

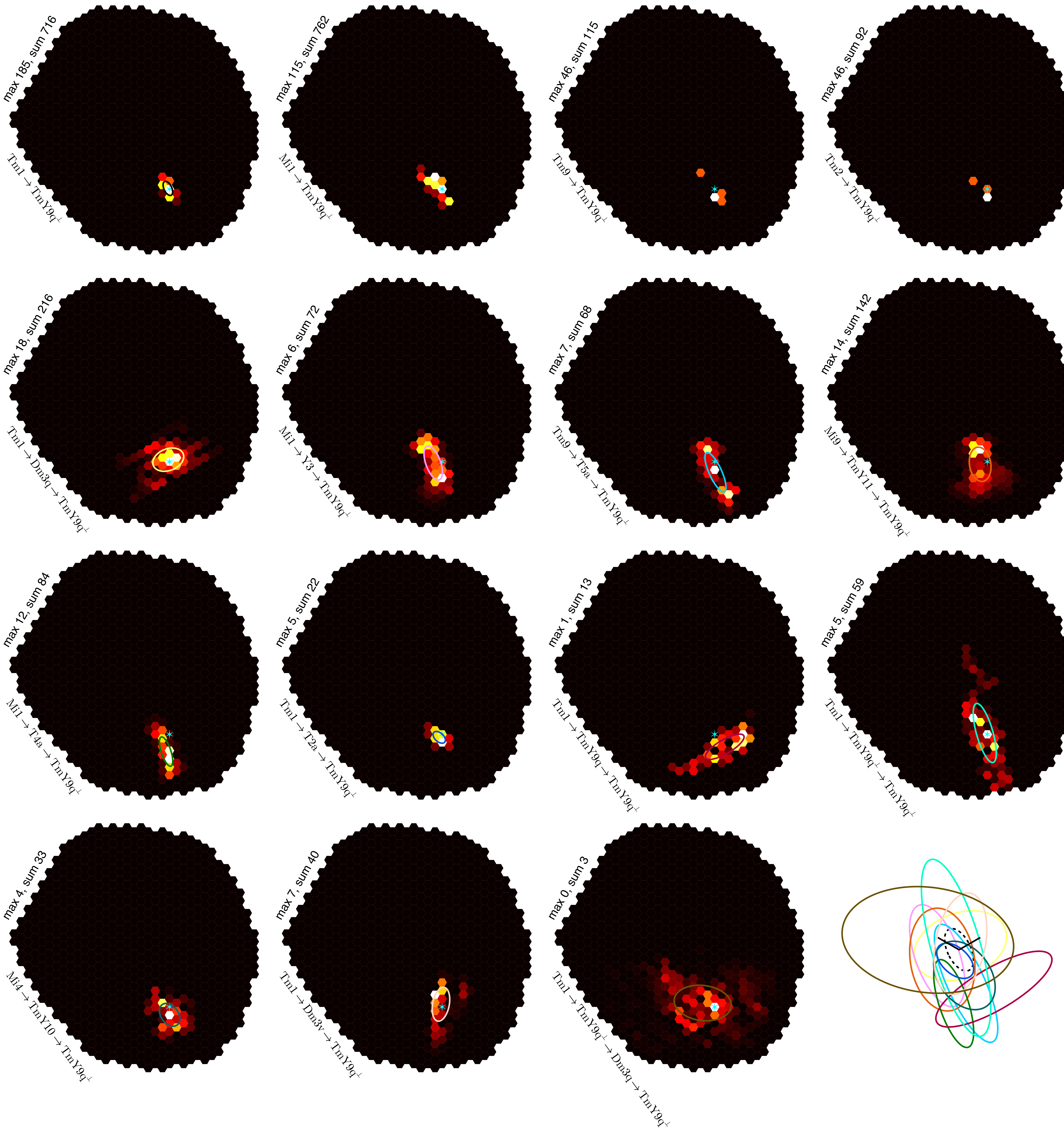
max 185, sum 716
Tm1 → TmY9q⊥
max 115, sum 762
Mi1 → TmY9q⊥
max 46, sum 115
Tm9 → TmY9q⊥
max 46, sum 92
Tm2 → TmY9q⊥
max 18, sum 216
Tm1 → Dm3q → TmY9q⊥
max 6, sum 72
Mi1 → Y3 → TmY9q⊥
max 7, sum 68
Tm9 → T5a → TmY9q⊥
max 14, sum 142
Mi9 → TmY11 → TmY9q⊥
max 12, sum 84
Mi1 → T4a → TmY9q⊥
max 5, sum 22
Tm1 → T2a → TmY9q⊥
max 1, sum 13
Tm1 → TmY9q → TmY9q⊥
max 5, sum 59
Tm1 → TmY9q⊥ → TmY9q⊥
max 4, sum 33
Mi4 → TmY10 → TmY9q⊥
max 7, sum 40
Tm1 → Dm3v → TmY9q⊥
max 0, sum 3
Tm1 → TmY9q⊥ → Dm3q → TmY9q⊥
